# Research-Practice-Collaborations Addressing One Health and Urban Transformation. A Case Study: Commentary on “Research-Practice-Collaborations in International Sustainable Development and Knowledge Production—Reflections from a Political-Economic Perspective”

**DOI:** 10.1057/s41287-022-00553-x

**Published:** 2022-07-18

**Authors:** Ana Maria Perez Arredondo

**Affiliations:** 1 Center for Development Research (ZEF), University of Bonn, Bonn, Germany; 2 International Centre for Sustainable Development (IZNE), University of Applied Sciences Bonn-Rhein-Sieg, Sankt Augustin, Germany

**Keywords:** Transdisciplinary research, One Health implementation, One Health doctoral training

## Abstract

One Health is an integrative approach at the interface of humans, animals and the environment, which can be implemented as Research-Practice-Collaboration (RPC) for its interdisciplinarity and intersectoral focus on the co-production of knowledge. To exemplify this, the present commentary shows the example of the Forschungskolleg “One Health and Urban Transformation” funded by the Ministry of Culture and Science of the State Government of Nord Rhine Westphalia in Germany. After analysis, the factors identified for a better implementation of RPC for One Health were the ones that allowed for constant communication and the reduction of power asymmetries between practitioners and academics in the co-production of knowledge. In this light, the training of a new generation of scientists at the boundaries of different disciplines that have mediation skills between academia and practice is an important contribution with great implications for societal change that can aid the further development of RPC.

## Résumé

« Une Santé» (en anglais: One Health) est un approche intégratif situé à l’interface entre les humains, les animaux, et l’environnement, qui peut être implémenté tel qu’une collaboration entre recherche et pratique (CRP) grâce à son interdisciplinarité et son accent sur la cocréation du savoir. Pour illustrer ce point, cet article prend le Forschungskolleg « Une Santé et Transformation Urbaine» (en anglais: One Health and Urban Transformation) financé par le Ministère de la Culture et Sciences du gouvernement du Nord-Rhin Westphalie en Allemagne. D’après nos analyses, les facteurs identifiés comme soutenant une meilleure implémentation du CRP pour le programme One Health sont ceux qui permettent une communication constante et une réduction des asymétries causés par le pouvoir entre les praticiens et les entités académiques dans la cocréation du savoir. Sur ce point, l’éducation d’une nouvelle génération de scientifiques, à l’intersection des différentes disciplines et avec des fortes aptitudes à la médiation entre la pratique et le monde académique, est une contribution important avec des grandes implications pour le changement sociétal, et qui peut en outre soutenir le développement du CRP.

## Introduction

This commentary adds to the reflections on research-practice collaborations (RPC), its perceptions, potentials, and shortfalls in the context of One Health (OH), an integrative approach that looks at the interconnections between the health of humans, animals and the environment. Moreover, a case example is analysed to provide empirical evidence of how specific factors—i.e. inputs, processes, outputs, and institutions—can be used for evaluating RPC (Bender 2022; Pärli 2022; Hansson and Polk 2018; Guimarães et al. 2019). The case presented is the Forschungskolleg “One Health and Urban Transformation” (FOH) funded by the Ministry of Culture and Science of the State Government of Nord Rhine Westphalia in Germany with the mandate to promote transdisciplinary research for developing solutions to the complex problems of the 21st century, and emphasizing the practical implementation of research results (Ministerium für Kultur und Wissenschaft des Landes Nordrhein-Westfalen 2022).

The structure of this commentary article is as follows, Sect. 2 introduces the audience to the OH approach and gives an overview of how OH relates to RPCs, the different understandings of OH, its potential, and the main criticism. After that, in Sect. 3, a description of the configuration of the FOH is given, looking closely at the roles of the partners, the collaboration process, and the practical applications. The learnings and pitfalls of the FOH can be found in Sect. 4.

## The One Health Approach as Research-Practice-Collaboration

“One Health” is a unifying concept at the intersection of humans, animals, and the environment (Zinsstag et al. 2005). The roots of OH, namely the recognition of health interdependencies between living organisms and their ecosystem, trace back to ancient writings and medical treatises, which gained renewed attention during the enlightenment period for guiding research on diseases transmittable from animals to humans, later evolved towards the modern health approaches in the 21st century when the grounds for comparative medicine were established, and finally fuelled the “One Medicine” notion for public health in the 20th century, focussed on the human–animal interactions (Perez Arredondo et al. 2021; Bruchhausen 2019). At the beginning of the 21st century, the “One Medicine” notion transitioned to OH after the recognition of the role of the environment in the health interdependencies between humans and animals.

The OH approach has gained attention in recent years as changes in climate, biodiversity, population, food systems, and globalization patterns are linked to the emergence and re-emergence of pandemic and epidemic-prone diseases that require close collaborations of different sectors and disciplines to be addressed. As an RPC, as defined by Bender in terms of *“any research activity that proactively includes practitioners at any given stage of research”* (Bender 2022), the OH approach is located at the boundary of science and society in the co-creation of knowledge and its applications differ by the levels of collaboration, the goals pursued, and the values and expertise of the actors involved (Galaz et al. 2015; Yasobant et al. 2019). Nowadays, OH became an approach that combines different narratives. The object of the first narrative is an intersectoral–interdisciplinary collaboration that links different forms of knowledge to generate a “*holistic understanding of disease burden and disease ecology to inform decision-makers”* (Bardosh et al. 2017). The second narrative considers OH as a unifying approach used for institutional change to address health problems (Zinsstag et al. 2005; Hitziger et al. 2018). Finally, the third narrative which is known as the extended OH originated in the transition from “One Medicine” to “One Health”, it includes a complex system perspective to address the ecological, social and political roots of OH problems, while at the same time has set on the quest to balance the normative values of the health of humans, animals, and the environment (Perez Arredondo et al. 2021; Bardosh et al. 2017; Degeling et al. 2019; Coghlan and Coghlan 2018).

The diversity of OH research, actions and applications reflects the holistic nature of the approach. On the one hand, with a utilitarian orientation that seeks knowledge to inform decisions and create institutional changes to break disciplinary and sectoral silo structures. While on the other hand, the extended OH narrative has set the ground for addressing epistemological and ethical considerations to push the boundaries of knowledge concerning the linkages between ecosystem conservation, animal health, and human health. Nonetheless, relatively soon after the OH concept was coined, critics emerged as the practical OH applications were limited, and despite the existence of empirical evidence and literature on health interdependencies, the way to achieve intersectoral–interdisciplinary collaborations at the human–animal–environment interface was regarded as pure theory. At first, the critics emphasized the difficulty of translating OH into the real world (Bardosh et al. 2017), but as practical applications emerged, new critics addressed the lack of representation of community and social actors as academics and policymakers led the implementation of OH initiatives. These later critics adhere to the fact that pure utilitarian views of OH actions are still dominating research and practice, as Schmiege et al. show in their review of OH in the context of coronavirus outbreaks (Schmiege et al. 2020), where an important share of the OH actions identified have the goal of avoiding the spread of disease from animals to human populations, controlling disease outbreaks in humans and economically important animal species, or monitoring the concentration of pathogens in the environment that represent risks to human health.

### Co-production of Knowledge Following the One Health Approach

The historical development of OH is a reflection of the evolution of collaborative efforts to generate and apply knowledge inside and out of academia. Therefore, it is of no surprise that a relatively new approach as OH created a division of opinions regarding the implications for knowledge generation and its practical applications, especially regarding the ethical considerations and epistemological developments needed to define and evaluate the health and well-being of nonhumans. In this background, methodological approaches, collaboration strategies, and action programmes to translate OH research into practice flourished with the ongoing movement for operationalizing OH, i.e. “*making the OH concept measurable and understandable”* (Perez Arredondo et al. 2021). It is of relevance that these new developments in the academic literature build on practical OH interventions at different levels, for instance, programmes comprising the assessment of the health burden and cost of zoonotic diseases as well as the implementation of vaccination campaigns to reach simultaneously animals and people (Bechir et al. 2004; Daugla et al. 2004; Schelling et al. 2004, 2007; Wyss et al. 2004*).* Those programmes proved valuable for all parties involved as the scientific knowledge of the diseases pathology, epidemiology, and aetiology was enriched with practical information on the ecologies of disease and the local public health system, and tangible outputs for the communities were generated.

The experiences and lessons that emerged from different OH initiatives made it possible to develop further methodologies, collaboration strategies, and definitions, and breach normative differences to address the epistemic and ethical dimensions of OH (Johnson and Degeling 2019).

## The Constellation of the Forschungskolleg One Health and Urban Transformation

As one of the numerous initiatives created to aid the development of the OH approach as RPC, the FOH was initiated in 2016 to address health problems in the context of urban transformation. The FOH is one of twelve collaborative research groups funded by the Ministry of Culture and Science of the State Government of North-Rhine Westphalia in Germany with the mandate to promote transdisciplinary research for the development of solutions to the complex problems of the 21st century, emphasizing the practical implementation of research results (Center for Development Research 2022). After the planning phase in 2016, the first cohort of doctoral students was admitted in 2017 as part of phase one of the project, and the second cohort joined in 2021 for starting phase two. A total of 25 doctoral students have been involved in the FOH.

The following sub-section describes the RPC process underlying the following three components, (1) problem identification and structuring, (2) problem analysis, and (3) implementation, and application (Jahn et al. 2012; Lawrence et al. 2022).

### Problem Identification and Structuring

The research topics of the FOH are organized around three broad thematic clusters that reflect the disciplinary expertise of the body of academic supervisors and the priorities of the implementation partners. The first thematic cluster, OH governance, is concerned with institutional developments, OH implementation and OH education capacities. The second cluster is food systems, emphasizing dietary behaviour, food production resources, and threats to food production like zoonotic diseases and antimicrobial resistance. The third cluster looks at land-use and land-use change, specifically in urban blue and green spaces.

**Fig. 1:**
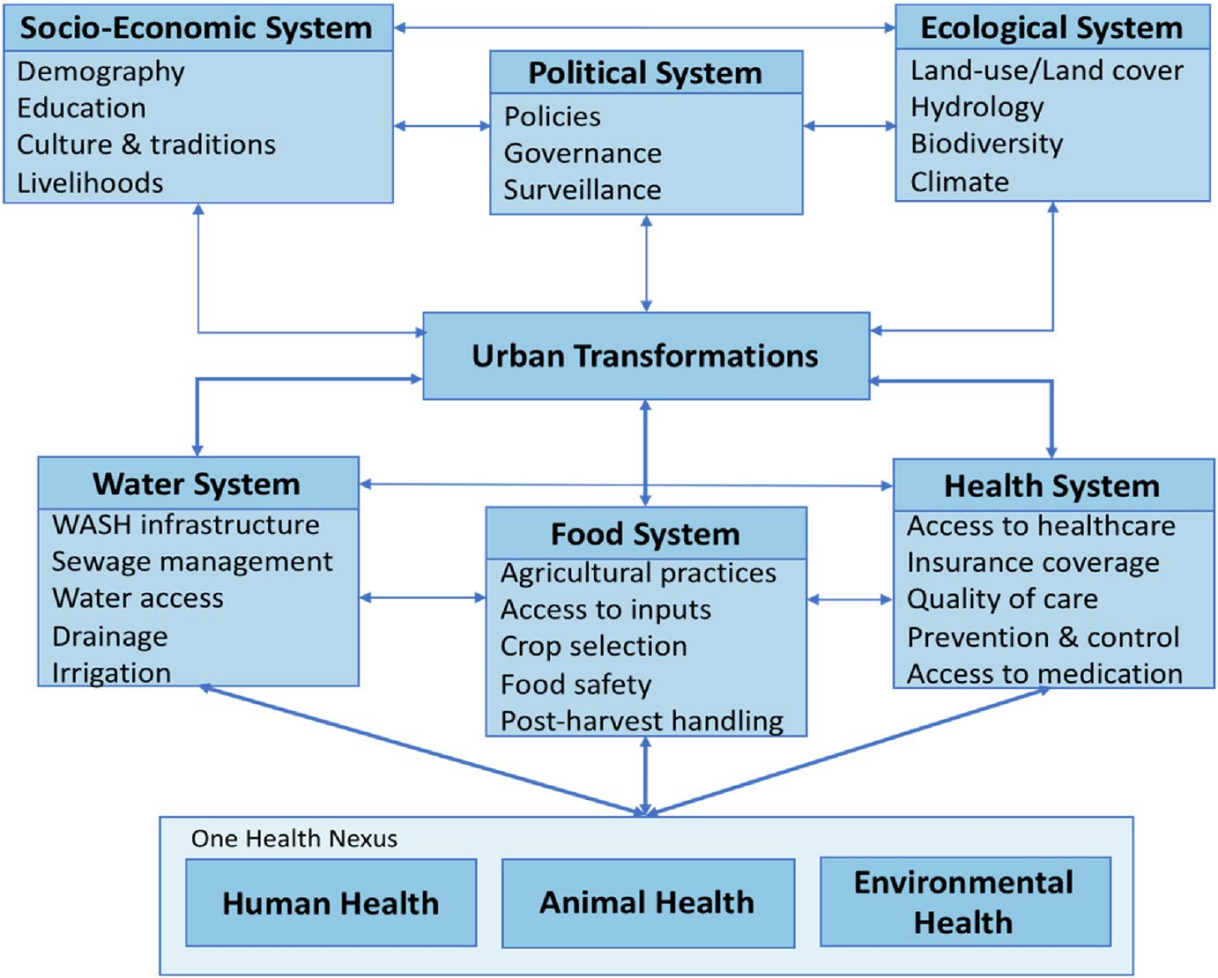
Framework for the work of the “Forschungskolleg One Health and Urban Transformation”. Depiction by Dr Timo Falkenberg (Falkenberg 2020)

The FOH works in the city context as different processes of global change that affects health take place in urban centres, like the growth of population and density, changes in the use of soil and water, and increasing demand for food. Urban centres are places subject to artificial conditions, which on the one hand, create externalities that directly affect the environment and create changes in the pathogenic interactions between living organisms. On the other hand, the fabricated circumstances may also favour salutogenic conditions as services and infrastructure are available to care for health and pursue welfare. Under these circumstances, the work of the FOH deals with urban transformation following a OH approach, as a way of addressing the complexity of health in Accra, Ahmedabad, Sao Paulo and the Ruhr Metropolis, which represent a big diversity in terms of economy, climatic conditions, culture, density, and size, used to generate knowledge and understandings on the systems connected to different processes of global change helpful to understand how different systems interact at the interface of human–animal–environment (see Fig. 1).

The consortium of the OHF is comprised of an awarding body, a coordinating body, and an implementation body. The awarding body is the University of Bonn, in which researchers are affiliated as doctoral students in the faculties of Agriculture, Matematics and Natural Sciences, or Medicine. The coordinating body is constituted by the Centre for Development Research (ZEF) of the University of Bonn, the International Centre for Sustainable Development (IZNE) of the University of Applied Sciences Bonn-Rhein-Sieg, and the Institute for Environment and Human Security (EHS) of the United Nations University, where the academic and the administrative coordination teams are nested alongside with a body of professors that supervise and guide the specific research topics. The implementation body consists of the partner research institutes in the different working areas, which are the Indian Institute of Public Health Gandhinagar (IIPHG) as part of the Public Health Foundation of India, the Institute of Statistical Social and Economic Research (ISSER) of the University of Ghana, and the Schools of Economics, Management, Accounting and Actuarial Sciences (FEA) and Pharmaceutical Sciences (FCF) of the University of Sao Paulo. The implementation partners carry out the activities of informing and evaluating the relevance of the research topics for the study areas, creating nexus with decision-makers and representatives of the research communities to be involved in the different research projects, and hosting the doctoral students for conducting fieldwork activities (Falkenberg 2020).

### Problem Analysis

**Fig. 2:**
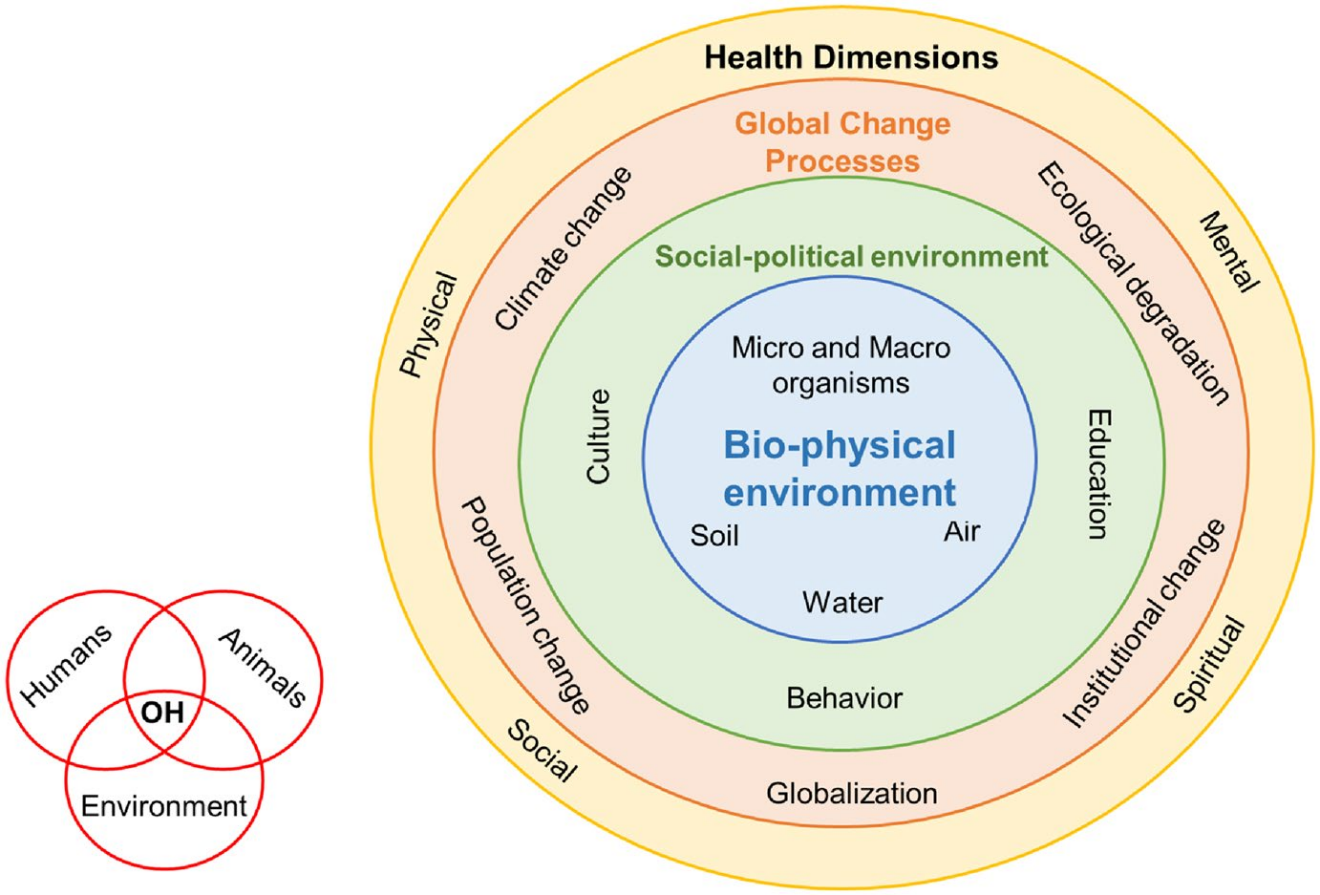
Framework for Research in One Health and Urban Transformation as a result of the participatory workshop for developing research and practice frameworks conducted in January 2020 in Bonn

Each doctoral student is in charge of an individual research project within the FOH, addressing a different research problem from the perspective of the discipline of expertise and interest of the student. The bridging of disciples is pursued by providing the students with interdisciplinary training and access to tutors and academic supervisors with diverse academic backgrounds that can aid the research process. Moreover, different frameworks for research and practice were created to provide a unified OH perspective for the FOH and align the research problems under one umbrella to communicate with practitioners. Figure 2 shows one of the frameworks created, where the starting point is the human–animal–environment interface.

As presented in Fig. 2, the FOH placed the bio-physical environment at the centre of interactions, where the living organisms need specific environmental and ecosystem conditions to live and interact, like water, oxygen, and minerals. The biophysical environment is ruled by a social–political environment, which has great implications for defining the patterns of global change that affect, both positively and negatively, the different dimensions of health and well-being. Each of the research projects can be located at a different intersection of the framework in Fig. 2.

### Implementation and Application

From 2016 to 2020, the FOH managed to establish collaborations in the four research areas with local actors through personal networks, engagement workshops, recurrent meetings for evaluating the relevance of the research topics, and the reporting of results through policy briefs. The main function of the local actors, besides giving feedback on the relevance of the research topics pursued, was to provide data used by the doctoral students to analyse the work of institutions around health, to assess the determinants of health-related to space use and space variation, to map the disease environments and dynamics, to look at food production and dietary patterns, among others. Notwithstanding, one of the main challenges for the inclusion of partners outside of academia was the perception of the project as an exogenous and purely academic exercise with limited implications for the practitioners.

As a result of the concerns expressed by the practitioners, the second phase of the project was set to emphasize research on the subject of food systems and OH implementation to balance the interests of the local partners and the academic body. The next section mentions briefly some of the main challenges faced and the learnings extracted.

## Learnings Extracted and the Way Forward

### Learnings

For this sub-section, a list of factors comprising input, process, outputs, and institutions for RPC (Pärli 2022) is used and is presented in Table 1. These factors are relevant for the discussion of the dynamics of social interactions within RPC following the work of Bender (Bender 2022).

**Table 1:** Factors to describe RPC in the context of the FOH

Input	Academic expertise: 20 supervisors affiliated either with the faculties of Agriculture, Mathematics and Natural Sciences, or Medicine of the University of Bonn, the International Centre for Sustainable Development (IZNE) of the University of Applied Sciences Bonn-Rhein-Sieg, or the Institute for Environment and Human Security (EHS) of the United Nations University
	Transnational cooperation institutes: Indian Institute of Public Health Gandhinagar as part of the Public Health Foundation of India, the Institute of Statistical Social and Economic Research (ISSER) of the University of Ghana, and the Schools of Economics, Management, Accounting and Actuarial Sciences (FEA) and Pharmaceutical Sciences (FCF) of the University of Sao Paulo
	Diversity of the team: research areas in 4 countries. Activities led by 25 doctoral students coming from 12 different countries and different professional backgrounds
	Motivation: addressing health and urban transformation; postgraduate training in applied research
	Qualifications: graduate students, with strong disciplinary focus and capacities to communicate with other disciplines
	Resources (students): experience in research, contact with local practitioners Resources (academia and donor): funding and research expertise and infrastructure
	Understanding: an underlying framework for One Health and Urban Transformation
	Previous contact: the academic institutions involved have a long-standing relationship and numerous joint projects
Process	Exchange and communication: constant bi-lateral communication between students and the coordination body, and the students and the supervisors. Moreover, once per year representatives of the donor institution, coordination body, supervisory board, and the implementation body meet to evaluate the activities and strategic planning
	Adaptive structure: there is little room for adjustments in terms of the thematic areas, roles and resources
	Co-creation: The students are working in close collaboration with practice partners and encouraged to engage with other students to align research objectives and have possibilities for comparing study sites. Unfortunately is limited to disciplinary compatibility and timing during the doctoral training
	Participatory tools and stakeholder-led activities: Stakeholders engagement workshops and evaluations
	Training: Interdisciplinary courses
	Available time: 3 years of funding for individual doctoral research projects
Output	Produced knowledge inside academia: doctoral thesis, scientific publications, seminar series in One Health
	Produced knowledge outside academia: policy briefs, frameworks for OH implementation
	Future collaboration perspectives: policy evaluations and training programmes in the research areas, as well as the scaling of the blueprints for evaluating OH programs in other regions
Institutions/constraints	Administration and academic supervision: University Bonn, Hochschule Bonn-Rhein-Sieg, United Nations University
	Funding: Ministry of Culture and Science of the State North-Rhine Westphalia
	Priorities: Health, urban transformation, research with practical applications
	Academic culture: Long-standing disciplinary expertise with interdisciplinary collaborations

First, if we look at the inputs for the RPC, it can be said that the FOH has a strong team of researchers affiliated with different institutes with a long-standing experience in research and policy evaluation in the selected research areas. Moreover, the contributions expected by each of the parties were clear, the understanding of OH was achieved through the joint development of a framework for research and practice, while the motivation of participating in the FOH was linked to the expertise obtained by addressing OH and Urban Transformation in the context of an RPC. This description is in line with the findings of Bender (Bender 2022) as the relevance for the work of the FOH is perceived to be directed to a better understanding of the local OH context. Nevertheless, this unified perception of values hints at a bigger problem, namely that the FOH was conceived and organized with academic actors as the centrepiece, making it difficult for practical partners to be actively engaged, take the co-supervision roles, or have tailored-made recommendations to inform decision making.

Regarding the process, the communication and co-creation of knowledge happened with more frequency among academics and less between academics, the practice partners, and the implementing body, creating what Bender described as *elite capture* (Bender 2022). This is the result of the strong academic background of the FOH, where the work in the research areas is condensed during the data collection period, while for the research planning and the data analysis processes the academic expertise tends to be prioritized to produce outputs with scientific relevance for achieving a doctoral degree. Fortunately, the increasing interest in OH by policymakers and other practitioners played an important role during the stakeholder engagement activities in the phase one of the FOH. Moreover, despite having a limited room for adjustments in terms of thematic areas, roles, and resources, the efforts and work of the doctoral students of phase two were focussed around the topics of food systems and OH implementation, that allowed for allocating the action of the local and practice partners intrinsically in the object of research, as shown in Fig. 2 in the “social-political environment” sphere, but also to work around one common understanding of the OH approach.

As for the outputs, it is difficult to report as the project is still ongoing. However, to mention but a few, phase one of the FOH, generated ample empirical evidence and publications in scientific journals. Moreover, five doctoral projects have been completed, and seminar series in “One Health and Urban Transformation” is offered for new doctoral students at the Centre for Development Research. Outside of academia, the most important outputs are different policy briefs and frameworks for the implementation of OH projects, with the perspectives of scaling the learnings to other areas that were part of the FOH focus.

### The Way Forward

To close this commentary, it remains to say that the challenges for RPC and the FOH are numerous; however, it is important to highlight that the time horizon in which the FOH operates made it possible for adjustments in specific factors that allow for balancing the power interactions between academics and practitioners, as allocating the priorities of practice partners intrinsically in the object of research and focussing further the thematic areas.

From the study case presented, the problem of RPC was less a policy-driven idealistic view of RPC as the concept of OH has enough traction on its own to be relevant for academic and non-academic actors, but issues of collective action were present at all stages. Looking at the impact of RPCs on social change, the main outcome of the FOH, disregarding the applicability and scalability of the research outcomes, is the training of a generation of scientists and professionals on interdisciplinary and transdisciplinary approaches. This new generation of scientists that work at the scientific boundaries and have mediating skills can avoid issues like the one of the FOH being planned by institutional discipline-based organizations, but also change how knowledge is produced, applied, and how RPC is implemented.
